# Dispositional Optimism and Context Sensitivity: Psychological Contributors to Frailty Status Among Elderly Outpatients

**DOI:** 10.3389/fpsyg.2020.621013

**Published:** 2021-01-13

**Authors:** Alberto Sardella, Vittorio Lenzo, George A. Bonanno, Gabriella Martino, Giorgio Basile, Maria C. Quattropani

**Affiliations:** ^1^Department of Clinical and Experimental Medicine, University of Messina, Messina, Italy; ^2^Department of Social and Educational Sciences of the Mediterranean Area, University for Foreigners “Dante Alighieri” of Reggio Calabria, Reggio Calabria, Italy; ^3^Department of Clinical Psychology, Teachers College, Columbia University, New York, NY, United States; ^4^School and Unit of Geriatrics, Department of Clinical and Experimental Medicine, University of Messina, Messina, Italy

**Keywords:** clinical psychology, psychological resilience, dispositional optimism, context sensitivity, elderly, frailty

## Abstract

The association of resilience-related factors with frailty is a recent research topic. Dispositional optimism and context sensitivity are two psychological factors that differently contribute to individual resilience. This study aimed at investigating whether dispositional optimism and context sensitivity might contribute to a multifactorial model of frailty, together with established relevant factors such as cognitive and physical factors. This cross-sectional study involved 141 elderly outpatients (42 males and 99 females) aged ≥65 years, who were referred to the Geriatrics and Multidimensional Evaluation Clinic of the University Hospital of Messina. We used the following measures: the Mini-Mental State Examination (MMSE) to screen for global cognitive functioning; 4-m gait speed and handgrip strength to measure physical performance; a 35-item Frailty Index (FI) to evaluate patients’ frailty status; the revised Life Orientation Test (LOT-R) to gauge dispositional optimism; and the Context Sensitivity Index (CSI) to measure context sensitivity. We found that LOT-R (*β* = −0.190, *p* = 0.038), CSI (*β* = −0.191, *p* = 0.035), and MMSE (*β* = −0.466, *p* < 0.001) were all significantly associated with FI. Gait speed was only marginally associated with FI (*β* = −0.184, *p* = 0.053). The present study showed a novel association of dispositional optimism and context sensitivity with frailty among elderly outpatients. These preliminary findings support a multidimensional approach to frailty in which even peculiar psychological features might provide a significant contribution.

## Introduction

The complex and joint interaction between different bio-psycho-social factors is a distinctive trait of aging trajectories. One challenge with elderly subjects is finding effective strategies that favor a positive adaptation to different age-related outcomes (Castelnuovo et al., 2015; Van Houtum et al., 2015; Yoo and Ryff, 2019). Consistently, several studies have shown that the maintenance of a healthy psychological state can be beneficial for reducing distress not only among subjects with psychopathological problems (Marchetti et al., 2019; Rosa et al., 2019; Vicario et al., 2019) but also among patients with chronic medical conditions (Di Giuseppe et al., 2018, 2019, 2020; Quattropani et al., 2018b; Martino et al., 2020a). Similarly, several studies have recently highlighted the importance of clinical psychological features in handling the consequences of age-related medical conditions for both patients (Quattropani et al., 2018a; Catalano et al., 2019, 2020; Kelly et al., 2019; Marchi et al., 2019; Martino et al., 2020c) and health professionals (Quattropani et al., 2017; Conversano et al., 2020).

In the context of aging, frailty represents one of the most compelling outcomes since several factors throughout the life course contribute to the severity of this condition. Frailty has been defined as increased vulnerability to stressors due to reduced homeostatic reserves (Clegg et al., 2013), and it has been broadly investigated in community (Morley et al., 2012) and clinical settings (Basile et al., 2019). From a theoretical perspective, several approaches have been proposed to characterize the construct of frailty. Two of the most representative models are the frailty phenotype model (Fried et al., 2001) and the deficit accumulation model (Rockwood and Mitnitski, 2007). The *frailty phenotype model* describes mainly a physical frailty, defined as the presence or absence of weight loss, fatigue, reduced gait speed, poor handgrip strength, and sedentary habits; consequently, patients are classified as robust, pre-frail, or frail. The *deficit accumulation model* proposes a multidimensional evaluation of frailty based on the weight of different age-related problems accumulated over time; in this model, frailty is measured using a Frailty Index (FI) calculated as the ratio between the deficits an individual presents and the number of age-related health variables considered in the evaluation.

The investigation of psychological features potentially associated with frailty is a recent topic of research. In light of the above-mentioned bio-psycho-social approach, frailty should be considered as a complex syndrome that affects not only biological processes but also psychological and social processes, leading to progressive adverse outcomes in old age (Gobbens et al., 2010). In line with this perspective, previous researches have explored different clinical psychological factors associated with frailty in both community populations and clinical settings. Accordingly, it has been suggested that depressive symptoms affect multidimensional frailty status in the community population, especially among women (Freitag and Schmidt, 2016). Moreover, loneliness, depression, and social isolation appear to be involved in the interaction between physical frailty and daily autonomy in community-dwelling older adults (Mulasso et al., 2016). Psychological factors, such as social and emotional support, resilience, and emotional well-being, have been suggested as potential protective factors for physical frailty in older subjects suffering from chronic medical conditions (Rubtsova et al., 2019; Yuan et al., 2020) and institutionalized older women (Furtado et al., 2020).

Psychological resilience is increasingly considered as a relevant factor contributing to individuals’ adaptation to several age-related challenges (Taylor and Carr, 2020). Dispositional optimism is commonly recognized as a psychological factor able to promote resilience and to promote a positive adaptation to aging. In accordance with the model originally proposed by Scheier and Carver, the human behavior is modulated by a stable dispositional feature, which is based on positive or negative expectations. Consistently, when the expectations are favorable, the goal-directed behavior is characterized by a significant effort by the individual; conversely, when the expectations are unfavorable, the individual exhibits less effort to overcome difficulties. In line with this theoretical framework, optimists tend to engage more in active coping strategies, when there are difficulties to overcome (Scheier and Carver, 1985). Dispositional optimism has been previously suggested as a psychological contributor of a better individual cardiovascular health, since subjects with higher levels of dispositional optimism tend more to adopt healthy behaviors, such as not smoking or engaging in physical activity (Serlachius et al., 2015). The positive role of optimism has also been discussed in the context of age-related clinical conditions, such as cognitive impairment (Dos Santos et al., 2018) and diabetes (Faghani et al., 2018). Additionally, higher levels of optimism have been associated with a better quality of life (QoL) among patients with heart failure (Kraai et al., 2018) and Parkinson’s disease (Gison et al., 2014).

Since elderly subjects frequently experience the need to adapt to new situations and challenges, the ability to read contextual cues and then flexibly regulate their behavior might be considered as an additional psychological resilience factor associated with aging. In accordance with the definition proposed by Bonanno et al. (2018), *context sensitivity* refers to individuals’ ability to accurately perceive their own emotional and physiological state, and react in appropriate ways to different life situations; therefore, it has been identified as a relevant factor of efficacious self-regulation Researchers have identified context sensitivity as a crucial factor involved in psychological adjustment and the onset of psychopathology following stressful life events (Coifman and Bonanno, 2010). Context sensitivity can be a beneficial factor for patients and caregivers; for example, it can help to prevent burnout syndrome among palliative care professionals (Lenzo et al., 2020a).

Frailty is a multidimensional syndrome, in which not only cognitive and physical factors but also psychological features may concur. Dispositional optimism is acknowledged as a psychological factor that able to promote resilient behaviors, with a consequent beneficial impact on individual health. The contribution of dispositional optimism has been discussed in the context of different chronic medical conditions; however, the association with frailty status among elderly subjects has not been investigated yet.

Context sensitivity is considered as a further resilience factor, which contributes to the individual adaptation and may explain how people differently cope with stressful events. The investigation of context sensitivity is novel in the context of elderly subjects, and in association with a negative age-related outcome as frailty.

In line with these considerations, the main purpose of the present study was to investigate interactions between frailty, dispositional optimism, and context sensitivity in a sample of elderly outpatients, in order to determine whether the two psychological factors might contribute to a multifactorial model of frailty, along with known contributors (e.g., cognitive and physical performances).

## Materials and Methods

### Participants

The present cross-sectional study involved elderly outpatients, referring to the Geriatrics and Multidimensional Evaluation Clinic of the University Hospital of Messina (Italy). Subjects with age ≥ 65 were evaluated for inclusion; the indicated range of age is consistent with the age of access to the geriatric clinics. The recruitment was carried out during the scheduled visits of the outpatients in the Clinic; each eligible outpatient participated in the study on a voluntary basis.

Each outpatient had to undergo a multidimensional evaluation, based on the assessment of cognitive status, physical performances and psychological functioning. In order to facilitate the comprehensive administration of the scales and the execution of the tasks, we included subjects without severe neurocognitive disorders, according to the DSM-5 diagnostic criteria (American Psychiatric Association, 2013), and/or severe functional and sensory limitations. Precisely, we excluded subjects with a Mini-Mental State Examination (MMSE) score ≤ 12; we additionally excluded subjects on wheelchairs and/or not able to walk, and subjects with severe limitations in the upper limbs; similarly, subjects with diagnosed severe visual and/or hearing impairments were excluded. We excluded patients with severe physical limitations also because the calculation of frailty status included the physical performances, besides other variables, as further explained in detail.

The main sociodemographic and clinical characteristics of the sample (*N* = 141) are reported in Table 1.

**Table 1:** Sociodemographic and clinical characteristics of the sample.

Patients (*N* = 141)	
*Sociodemographic*	
Age (years; mean ± SD)	80.31 ± 6.84
Gender Male (*n*, %) Female (*n*, %)	42 (29.8) 99 (70.2)
Education (years; mean ± SD)	7.09 (± 3.83)
Marital status Married (*n*, %) Widow/er (*n*, %) Other (*n*, %)	71 (50.4) 56 (39.7) 14 (9.9)
*Clinical*	
MMSE (mean ± SD)	22.61 (± 4.52)
FI (mean ± SD)	0.25 (± 0.11)
Frailty status Frail (*n*, %) Not frail (*n*, %)	71 (50.4) 70 (49.6)
LOT-R (mean ± SD)	18.20 (± 5.57)
CSI (mean ± SD)	18.94 (± 1.57)

SD, Standard Deviation; MMSE, Mini-Mental State Examination; FI, Frailty Index; LOT-R, Life Orientation Test-Revised; and CSI, Context Sensitivity Index.

### Ethics Statement

All procedures completed in the study were in accordance with the ethical standards of our institutional research committee and with the 1964 Declaration of Helsinki and its later amendments. Informed consent was obtained from all participants. The Ethics Committee of the University Hospital of Messina approved the protocol of this study (Prot. 23/19, University Hospital Ethics Committee).

### Measures

The evaluation protocol was developed in agreement with a senior geriatrician and a senior clinical psychologist; trained psychologists and trained physicians performed the assessments.

We used the MMSE to screen for global cognitive functioning (Folstein et al., 1975); the MMSE returns a score from 0 to 30, with higher scores corresponding to better performances. We adjusted the raw scores for age and education, in accordance with common normative data (Magni et al., 1996).

We measured physical performances by testing 4-m gait speed (expressed as meters per second) and handgrip strength (expressed in kilograms, measured by a Jamar dynamometer).

We evaluated the frailty status by the calculation of a 35-deficit FI, according to the standard procedure (Searle et al., 2008). The FI is expressed as a ratio of health-related deficits present to the total number of deficits considered; consistently, the greater the number of identified deficits, the higher the degree of frailty. Subjects with a FI ≥ 0.25 are commonly classified as frail (Rockwood and Mitnitski, 2007). The 35 variables that were evaluated for the calculation of the FI are provided as Supplementary Material.

We measured dispositional optimism using the Italian version of the revised Life Orientation Test (LOT-R; Scheier et al., 1994; Giannini et al., 2008). The LOT-R is a 10-item questionnaire based on a 5-point Likert scale ranging from “strongly disagree” to “strongly agree”; higher scores reflect a greater expectation of positive results.

We used the Context Sensitivity Index (CSI), a 20-item self-report questionnaire, to assess the patients’ ability to accurately identify cues to contextual demands across different hypothetical situations (Bonanno et al., 2018). The items are rated on a 7-point Likert scale ranging from 1 (not at all) to 7 (very much). The CSI measures individuals’ ability to capture the absence or presence of stressor context cues and calculates an overall CSI score by averaging the Cue Presence and Cue Absence indexes. We used the total CSI score for our observations; higher scores are an expression of a greater individual contextual sensitivity.

### Statistical Analysis

The data were analyzed using the statistical software IBM SPSS Statistics for Windows, Version 22.0 (Armonk, NY: IBM Corp.). We classified the subjects into frail and not frail groups according to their FI scores; subjects with scores of FI ≥ 0.25 were classified as frail. Differences between frail and not frail subjects were evaluating using the Student’s t test; the Chi-squared test was performed to calculate differences in the proportion of the variable “gender” among frail and not frail subjects; gender was categorized as follows: “0 = male; 1 = female.” Descriptive data were reported in terms of mean, standard deviation (SD), and percentage.

We performed univariate linear regressions to explore significant associations of the investigated variables with frailty. The multivariate linear regression model for frailty included the variables that were found significant at the univariate regressions. Precisely, the multivariate regression model was developed by hierarchically including the variables, as follows: we initially included the sociodemographic variables (e.g., age, gender, and education), then the clinical ones (e.g., global cognitive functioning and physical performances), since they are known contributors to frailty; ultimately, we tested the contribution of the novel psychological variables to explain the model.

Values of *p* < 0.05 were considered as statistically significant.

## Results

The study involved 141 elderly outpatients (42 males and 99 females), with a mean age of approximately 80 years. The patients exhibited mild-to-moderate cognitive impairments (MMSE mean score 22.6 ± 4.5) and showed a mean FI score of 0.25 (the FI scores ranged from 0.05 to 0.50).

The elderly outpatients classified as frail were significantly less educated than those classified as not frail (*p* = 0.012). Furthermore, the frail subjects exhibited significantly worse global cognitive (MMSE) and physical (handgrip and gait speed) performances than the not frail subjects (both *p* < 0.001). The psychological profile was also different between not frail and frail subjects, with the frail subjects showing lower levels of dispositional optimism (*p* = 0.001) and context sensitivity (*p* = 0.048). The main differences between the subjects according to their frailty status are summarized in Table 2.

**Table 2:** Main differences between frail and not frail patients.

	Not frail (*N* = 70)		Frail (*N* = 71)			
					*χ*^2^	*p*
Gender (m/f)	25/45		16/55		19.282	0.09
	**Mean**	**SD**	**Mean**	**SD**	***t***	***p***
Age	79.51	6.04	81.1	7.49	−1.383	0.16
Education	7.9	3.931	6.3	3.58	2.533	***0.012***
FI	0.16	0.04	0.34	0.06	−19.117	***<0.001***
MMSE	25.61	2.886	19.6	4.74	9.046	***<0.001***
Handgrip	19.966	6.5195	14.57	6.75	4.824	***<0.001***
Gait speed	0.7373	0.16304	0.55	0.17	6.212	***<0.001***
LOT-R	20.04	5.23	16.25	5.31	3.575	***0.001***
CSI	19.82	1.68	17.86	1.29	1.821	***0.048***

SD, Standard Deviation; MMSE, Mini-Mental State Examination; FI, Frailty Index; LOT-R, Life Orientation Test-Revised; and CSI, Context Sensitivity Index. Significant differences are reported in bold.

### Univariate and Multivariate Linear Regressions

We performed different univariate linear regressions with FI as the dependent variable, in order to investigate the association of our variables of interest with frailty. The analysis showed that age (*β* = 0.180, *p* = 0.03) and education (*β* = −0.168, *p* = 0.046) were both significantly associated with frailty. Additionally, MMSE (*β* = −0.637, *p* < 0.001), handgrip strength (*β* = −0.453, *p* < 0.001), and gait speed (*β* = −0.528, *p* < 0.001) were significantly associated with FI, as expected. Eventually, both the LOT-R (*β* = −0.319, *p* = 0.001) and the CSI (*β* = −0.343, *p* = 0.002) scores were significantly associated with FI. The univariate regressions are summarized in Table 3.

**Table 3:** Univariate linear regression for FI.

	B	SE(B)	*β*	*t*	*p*	95% CI	
						Lower	Upper
Age	0.003	0.001	0.180	2.154	***0.03***	0	0.005
Gender	0.034	0.019	0.148	1.759	0.08	−0.004	0.072
Education	−0.005	0.002	−0.168	−2.010	***0.046***	−0.009	0.001
MMSE	−0.015	0.002	−0.637	−9.732	***<0.001***	−0.018	−0.012
Handgrip	−0.007	0.001	−0.453	−5.986	***<0.001***	−0.009	−0.004
Gait speed	−0.292	0.040	−0.528	−7.284	***<0.001***	−0.372	−0.213
CSI	−0.022	0.007	−0.343	−3.179	***0.002***	−0.036	−0.008
LOT-R	−0.006	0.002	−0.319	−3.310	***0.001***	−0.009	−0.002

MMSE, Mini-Mental State Examination; FI, Frailty Index; LOT-R, Life Orientation Test-Revised; and CSI, Context Sensitivity Index. Significant values are reported in bold.

We computed a multivariate linear regression to identify the variables independently associated with frailty status and to understand whether our explored psychological indexes could contribute to explaining a multifactorial model of frailty, along with several other known factors. As previously stated, the multivariate regression was hierarchically developed, considering those variables that were found significant in the univariate analysis. In the first step of the model, we included the sociodemographic variables (i.e., age and education). In the second step, we included the cognitive and physical variables (i.e., MMSE, handgrip strength, and gait speed), representing the factors most commonly associated with frailty. In the third and final step of the model, we included the psychological indexes (i.e., LOT-R and CSI), our novel potential contributors to frailty. The findings of the multivariate regression are reported in Table 4.

**Table 4:** Hierarchical multivariate linear regression.

	*R*	*R*^2^	F change	*β*	*p*
*Step 1*	0.233	0.055	2.133		
Age				0.038	0.74
Education				−0.223	0.057
*Step 2*	0.676	0.457	17.558		
Age				0.015	0.86
Education				−0.070	0.45
MMSE				−0.536	***<0.001***
Handgrip				−0.071	0.48
Gait speed				−0.254	***0.009***
*Step 3*	0.719	0.516	4.228		
Age				−0.016	0.85
Education				−0.085	0.34
MMSE				−0.466	***<0.001***
Handgrip				−0.046	0.63
Gait speed				−0.184	0.053
LOT-R				−0.190	***0.038***
CSI				−0.191	***0.035***

MMSE, Mini-Mental State Examination; FI, Frailty Index; LOT-R, Life Orientation Test-Revised; and CSI, Context Sensitivity Index. Significant values are reported in bold.

The findings from the first step of the hierarchical model were not significant, accounting for only age and years of education (*R*^2^ = 0.055, *p* = 0.12). The findings from the second step, which additionally accounted for cognitive and physical factors, were statistically significant at *R*^2^ = 0.457; this step was substantially explained by the inclusion of MMSE (*β* = −0.536, *p* < 0.001) and gait speed (*β* = −0.254, *p* = 0.009) in the model. The findings from the inclusion of the two psychological indexes were globally significant (*R*^2^ = 0.516, *p* < 0.001). According to the final model, the LOT-R scores (*β* = −0.190, *p* = 0.038), CSI scores (*β* = −0.191, *p* = 0.035), and persistent MMSE scores (*β* = −0.466, *p* < 0.001) were all significantly associated with FI. Gait speed was only marginally associated with FI (*β* = −0.184, *p* = 0.053).

## Discussion

To the best of our knowledge, this is the first study to explore dispositional optimism and context sensitivity among elderly outpatients and to investigate their association with frailty status, as expressed by a calculated FI.

We investigated the contribution of the two psychological factors by developing a hierarchical regression model that accounted for different sociodemographic and clinical factors, which are widely acknowledged as predictors of patients’ trajectories toward frailty: age, years of education, and cognitive and physical functioning (Etman et al., 2012; Basile and Sardella, 2020; Sardella et al., 2020). Our findings, though preliminary, showed that in our evaluated sample of outpatients, besides the global cognitive functioning, additional psychological factors, namely dispositional optimism (measured through the LOT-R) and context sensitivity (measured through the CSI), were also significantly associated with frailty. Those subjects who exhibited a better health status (indicated by a lower FI score) also exhibited better global cognitive functioning, higher levels of dispositional optimism, and greater context sensitivity.

Dispositional optimism is an interesting psychological construct with multiple implications, which is able to promote the assumption of healthy behaviors. Previous evidence has highlighted the general positive association between adults’ optimism and physical health, and the beneficial role of optimism in the treatment of such chronic medical conditions as cardiovascular diseases, cancer, diabetes, and neurological pathologies (Rasmussen et al., 2009; Schiavon et al., 2017). We can reasonably assume that frailty is the result of the interaction between multiple factors and changes throughout the life course; therefore, our findings echo this general positive conceptualization of dispositional optimism, extending the evidence not only to a physical frailty but also to a multifactorial one.

Context sensitivity, within the theoretical framework proposed by Bonanno et al. (2018), has not been previously explored among elderly outpatients, or in association with frailty. Instead, this psychological construct has often been associated with mental health, since its protective role in the development of emotional disorders such as depression, anxiety, and complicated grief (Bylsma et al., 2008; Diminich and Bonanno, 2014; Harvey et al., 2014; Coifman et al., 2016). Recently, the positive contribution of individuals’ ability to respond flexibly to contextual cues has been showed in older adults, resulting beneficial for a better adaptation to pain, although the topic requires future clarifications (Flink et al., 2019). According to the theoretical framework behind context sensitivity, particular regulatory strategies are not necessarily beneficial or maladaptive; instead, the benefit lies in the flexible use of these strategies in response to environmental changes (Bonanno and Burton, 2013; Kobylińska and Kusev, 2019). As they progressively approach a condition of frailty, the elderly gradually lose multiple functions and skills, increasingly exposing themselves to negative outcomes. A hypothetical explanation of our results could be that those elderly who have more easily and flexibly adapted to these progressive age-related changes (e.g., the onset of disease, loss of autonomy, etc.) also exhibit a lower degree of frailty. Assuming that frailty is the result of multifactorial concurring variables (as expressed by the FI scores), our study suggests that context sensitivity might be considered as a further psychological factor that contributes to the elderly adaption to age-related challenges.

Living with chronic medical conditions adds a significant burden to individuals, so it is encouraging to find evidence that some psychological factors might help patients to manage their pathologies (Filippello et al., 2016; Gentili et al., 2019; Martino et al., 2019a,b, 2020b; Quattropani et al., 2019; Lenzo et al., 2020b; Vita et al., 2020). Aging involves the interaction among several bio-psycho-social variables that can concurrently influence patients’ trajectories from normal aging to disability. From this perspective, a progressively worsening frailty status increasingly exposes elderly subjects to a higher risk of disability (Makizako et al., 2015). Researchers have grown an increasingly interest in psychological resilience, even in the context of frailty, although the most common focus has been on physical frailty (Wong et al., 2020).

The findings of the current study are also in line with the general perspective of a multidisciplinary approach to patients with chronic medical conditions, which suggest that implementing protocols based on both psychological and physical interventions could be beneficial (Conversano, 2019; Martino et al., 2019b). Improving patients’ dispositional optimism and context sensitivity might be the novel target of mindfulness-based cognitive therapy (De Jong et al., 2016), peer-to-peer support (Callus and Pravettoni, 2018), and group therapies (Lo Coco et al., 2019), with the purpose of developing tailored psychological interventions in patients with chronic medical conditions (Conversano et al., 2019). The research in psychology is moving toward an increasingly patient-centered multidimensional approach, as recently debated within a psychodynamic perspective that highlighted the joint relevance of cognitive, emotional, and personality characteristics in the evaluation of clinical populations (Lingiardi et al., 2010; Hilsenroth et al., 2018; Lingiardi and McWilliams, 2018), with a peculiar concern on the link between aging and psychopathology (Del Corno and Kiosses, 2018).

The current study presents some limitations. The cross-sectional design did not allow a determination of causal relationships. Moreover, the study’s single clinical setting was an outpatient clinic, and this narrow focus might potentially reduce the generalizability of the findings. Finally, because of the relatively small number of subjects and the sample’s predominance of women, we could not fully explore gender differences between subjects. Longitudinal studies, involving larger samples, should be conducted to confirm these preliminary findings.

Despite the acknowledged limitations, this study offers some significant contributions. As mentioned, several studies involving community populations have employed Fried’s frailty phenotype to define frailty status. However, our addition of the deficit accumulation model (Rockwood and Mitnitski, 2007) and the associated FI scores provide a helpful strategy to capture the outpatients’ clinical complexity. While the frailty phenotype returns an immediate identification of the not-disabled elderlies’ risk of negative events, the FI provides a comprehensive assessment based on deficit accumulation (Cesari et al., 2014). Furthermore, for our unique observations in the context of frailty, we used the two most commonly shared tools, in order to accurately measure dispositional optimism and context sensitivity, in line with their respective theoretical frameworks and in line with previous studies.

## Conclusion

The present study highlights the novel association of dispositional optimism and context sensitivity with frailty among elderly outpatients. Dispositional optimism is defined as the individual tendency to expect positive outcomes from different challenges over the entire life span; it has been identified as a psychological factor affecting individuals’ health status. Similarly, the ability to accurately and sensitively perceive cues to contextual demands has been identified as a further significant component of successful self-regulation; consistently, it could help elderly patients to better adapt to challenges affecting their physical and mental health.

These preliminary findings represent a starting point for a multidimensional approach to frailty and an acknowledgment that even peculiar psychological features might play a significant role. One potential implication for both physicians and clinical psychologists could be that the Comprehensive Geriatric Assessment (CGA), the standard geriatric patient-centered methodology, could be enriched to better describe elderly outpatients’ complexity. Dispositional optimism and context sensitivity should be investigated in the future as potentially useful targets for designing psychological interventions for the elderly, focusing on improving or strengthening individual psychological factors.

## Data Availability Statement

The raw data supporting the conclusions of this article will be made available by the authors, without undue reservation.

## Ethics Statement

The studies involving human participants were reviewed and approved by the Ethics Committee of the University Hospital of Messina, Messina, Italy (Prot. 23/19). The patients/participants provided their written informed consent to participate in this study.

## Author Contributions

AS made significant contributions to the study design, the drafting of the manuscript, the statistical analyses, and the data interpretation. VL performed the statistical analysis, provided an additional interpretation of the data, and contributed to the manuscript draft. GAB and GM provided the significant conceptual contribution to the manuscript and revised it. GB and MQ critically revised the manuscript. All authors contributed to the article and approved the submitted version.

### Conflict of Interest

The authors declare that the research was conducted in the absence of any commercial or financial relationships that could be construed as a potential conflict of interest.
