# Supplementary material for: Dispositional Optimism and Context Sensitivity: Psychological Contributors to Frailty Status Among Elderly Outpatients
Source: Front Psychol. 2021 Jan 13;11:621013. doi: 10.3389/fpsyg.2020.621013 (PMC7838363; doi:10.3389/fpsyg.2020.621013)
Supplement: Supplementary file 1 [file Table_1.docx]

Supplementary Material

# Supplementary Table. Variables considered in the calculated Frailty Index (FI).

| Hospitalization | Pain | Urinary incontinence | Heart failure | Cerebrovascular disease |
| --- | --- | --- | --- | --- |
| Fractures | Bathing | Faecal incontinence | Chronic Obstructive Pulmonary Disease | Handgrip strength |
| Caregiver | Dressing | Telephone | BMI | Parkinsonism |
| Cognitive status | Walking | Drugs | Cancer | Gait speed |
| Malnutrition | Getting up / Sitting down | Hypertension | Cirrhosis | Medications |
| Dehydration | Feeding | Diabetes | Chronic kidney failure | Benzodiazepines |
| Oral health | Toileting | Heart disease | Obesity | Neuroleptics |
|  | | | ***Total number of detected deficits*** | ***Frailty Index*** |
|  |  |  | __ / 35 |  |

*Note:* The FI is expressed as a ratio of health deficits present to the total number of deficits considered; the greater the number of health deficits, the higher the degree of frailty. According to this approach, patients with a FI ≥0.25 are commonly considered frail.
